# Dr, Hering’s Tables of Throat, Chest and Cough Symptoms

**Published:** 1882-05

**Authors:** 


### AFFECTIONS OF THE THROAT.

| IN THE THROAT. | Aconite. | Arsenic. | Bella. | Bryonia. | Capsic. | Carb veg. | Caustic. | Cham. | China. | Cocculus. | Drosera. | Ferrum. | Hyosc. | Ignatia. | Lachesis. | Lycopod. | Merc. viv. | Nux vom. | Phosphor. | Phosph.acid | Puls. | Rhus. | Spigelia. | Spong. | Staphys. | Stram. | Sulphur. | Hep. Sulph. |
|---|---|---|---|---|---|---|---|---|---|---|---|---|---|---|---|---|---|---|---|---|---|---|---|---|---|---|---|---|
| Burning | — | — | — | ... | ... | — | ... | ... | ... | — | — | ... | — | ... | ... | ... | — | — | ... | ... | — | ... | ... | ... | ... | — | — | — |
| Pressure | ... | ... | ... | — | ... | — | — | ... | ... | ... | ... | — | ... | ... | ... | ... | — | — | ... | ... | — | ... | ... | ... | ... | ... | — | — |
| Pain | — | ... | — | ... | ... | — | ... | — | — | — | ... | ... | ... | ... | — | — | — | — | ... | — | — | — | ... | ... | ... | ... | — | — |
| Inflammation | — | — | — | — | — | — | ... | — | ... | ... | ... | ... | ... | ... | — | — | ... | ... | ... | — | — | — | ... | ... | ... | ... | — | ... |
| Swelling | — | ... | ... | — | ... | — | ... | — | ... | ... | ... | ... | ... | — | ... | ... | ... | ... | ... | ... | ... | — | — | ... | ... | ... | ... | ... |
| Feeling as if swollen | ... | ... | ... | — | ... | ... | — | ... | — | ... | ... | ... | — | ... | ... | ... | ... | — | ... | ... | — | ... | ... | ... | ... | ... | — | — |
| Feeling of dryness | ... | ... | — | — | ... | — | — | ... | ... | ... | — | ... | ... | ... | ... | — | — | ... | — | ... | — | ... | ... | ... | ... | — | — | — |
| Itching | ... | ... | ... | — | ... | ... | ... | ... | ... | ... | ... | ... | ... | ... | ... | ... | ... | — | ... | ... | ... | ... | — | ... | ... | ... | ... | ... |
| Scraping | — | — | ... | ... | ... | — | — | ... | — | ... | ... | ... | ... | ... | ... | ... | ... | — | ... | — | — | ... | ... | ... | — | ... | ... | — |
| Soreness | — | — | — | — | ... | — | ... | ... | — | — | — | ... | — | — | ... | ... | — | — | ... | — | — | ... | ... | ... | — | ... | ... | — |
| Difficult swallw'g | — | ... | ... | — | ... | ... | ... | — | ... | ... | ... | ... | — | — | ... | — | — | — | ... | — | — | — | ... | — | ... | — | ... | — |
| Painful do | — | ... | ... | — | — | ... | ... | — | — | — | ... | — | ... | ... | ... | ... | ... | — | ... | — | — | — | — | ... | — | — | ... | ... |
| Stitches | ... | ... | — | — | — | — | ... | ... | — | ... | ... | ... | ... | — | ... | ... | ... | — | — | — | — | ... | — | — | — | ... | — | — |
| Ulcers | ... | ... | — | ... | — | — | ... | ... | ... | ... | ... | ... | ... | ... | — | — | — | ... | ... | — | ... | ... | ... | — | ... | ... | ... | — |
| H'wk'g up phle'm | ... | ... | — | — | ... | ... | — | — | ... | ... | ... | ... | — | ... | — | — | ... | — | ... | ... | ... | — | — | ... | — | ... | ... | — |
| Increased secretion of phlegm | ... | ... | — | ... | ... | — | — | — | — | ... | ... | ... | ... | — | — | — | — | — | — | ... | — | — | — | ... | — | ... | ... | — |
| a) SYMPTOMS WORSE. | | | | | | | | | | | | | | | | | | | | | | | | | | | | |
| In the morning | — | ... | ... | — | ... | ... | — | ... | — | ... | ... | ... | ... | — | ... | ... | ... | — | — | ... | — | ... | ... | ... | ... | ... | — | — |
| In the forenoon | ... | ... | ... | ... | ... | ... | ... | ... | ... | ... | ... | ... | ... | ... | ... | ... | ... | ... | — | ... | ... | ... | ... | ... | ... | ... | ... | ... |
| In the afternoon | ... | ... | ... | ... | ... | ... | ... | — | ... | ... | — | ... | ... | ... | ... | ... | ... | — | — | ... | ... | — | ... | ... | ... | ... | ... | ... |
| In the evening | ... | ... | ... | — | ... | — | — | ... | ... | ... | ... | — | ... | — | — | ... | ... | — | ... | ... | — | ... | ... | ... | — | ... | ... | ... |
| At night | ... | ... | ... | — | ... | — | — | ... | ... | ... | ... | — | ... | ... | — | ... | ... | — | — | ... | — | ... | ... | — | ... | ... | ... | ... |
| After midnight | ... | ... | ... | ... | ... | ... | ... | ... | ... | ... | ... | ... | ... | ... | ... | ... | ... | — | ... | ... | — | ... | ... | ... | ... | ... | ... | ... |
| b) SYMPTOMS AGGRAVATED. | | | | | | | | | | | | | | | | | | | | | | | | | | | | |
| By breathing | ... | ... | ... | ... | ... | ... | ... | ... | — | ... | ... | ... | ... | ... | ... | ... | ... | ... | ... | ... | ... | ... | ... | ... | ... | ... | ... | ... |
| By touching | ... | ... | ... | — | ... | ... | ... | ... | ... | ... | ... | — | ... | ... | — | ... | ... | ... | ... | ... | ... | ... | ... | ... | — | ... | ... | ... |
| After overheating | ... | ... | ... | — | — | ... | ... | ... | ... | ... | ... | ... | ... | — | ... | ... | ... | ... | ... | ... | ... | ... | ... | ... | ... | ... | ... | ... |
| After taking cold | ... | ... | ... | — | ... | ... | ... | — | ... | ... | ... | ... | — | ... | ... | ... | ... | — | ... | ... | ... | — | ... | ... | ... | ... | ... | ... |
| When eating | ... | ... | ... | ... | ... | ... | ... | ... | ... | ... | ... | — | ... | — | ... | ... | ... | — | ... | ... | ... | ... | ... | — | ... | ... | ... | ... |
| When drinking | ... | ... | — | — | ... | ... | ... | ... | ... | ... | ... | ... | — | ... | ... | ... | ... | ... | ... | ... | ... | ... | ... | ... | ... | — | ... | ... |
| After eating | ... | ... | ... | — | ... | ... | ... | — | — | ... | ... | ... | ... | — | ... | ... | ... | — | ... | ... | ... | — | ... | — | ... | ... | ... | ... |
| When coughing | ... | ... | ... | ... | — | ... | ... | ... | ... | ... | ... | ... | ... | ... | ... | ... | ... | ... | ... | ... | ... | ... | ... | ... | ... | ... | ... | ... |
| When lying down | ... | ... | ... | ... | ... | ... | ... | — | ... | ... | ... | ... | ... | ... | ... | ... | ... | — | ... | ... | ... | — | ... | ... | ... | ... | ... | ... |
| When inhaling cold air | ... | ... | ... | ... | ... | ... | ... | ... | ... | ... | ... | ... | ... | ... | ... | ... | ... | — | ... | ... | ... | ... | ... | ... | ... | ... | ... | ... |
| When swallowing | — | ... | — | — | — | ... | ... | — | — | — | ... | — | ... | ... | ... | ... | — | — | ... | ... | — | — | — | ... | ... | — | — | — |
| When talking | — | ... | ... | ... | ... | ... | ... | ... | ... | ... | ... | ... | ... | ... | ... | ... | ... | ... | ... | ... | — | ... | ... | ... | — | ... | ... | ... |
| c) SYMPTOMS BETTER. | | | | | | | | | | | | | | | | | | | | | | | | | | | | |
| After rising | ... | ... | ... | ... | ... | ... | ... | ... | ... | ... | ... | ... | ... | ... | ... | ... | ... | — | ... | ... | ... | ... | ... | ... | ... | ... | ... | ... |
| After drinking | ... | ... | ... | ... | ... | ... | ... | ... | ... | ... | ... | ... | ... | ... | ... | ... | ... | ... | ... | ... | ... | ... | ... | ... | ... | ... | ... | ... |
| When moving | ... | ... | ... | ... | ... | ... | ... | ... | ... | ... | ... | ... | ... | ... | ... | ... | ... | ... | ... | ... | — | ... | ... | ... | ... | ... | ... | ... |
| When eating | ... | ... | ... | ... | ... | ... | ... | ... | ... | ... | ... | ... | ... | — | ... | ... | ... | — | ... | ... | ... | ... | ... | ... | — | ... | ... | ... |
| When drinking | ... | ... | ... | — | ... | ... | ... | ... | ... | ... | ... | ... | ... | ... | ... | ... | ... | — | ... | ... | ... | ... | ... | ... | ... | ... | ... | ... |
| When swallowing | ... | ... | ... | ... | ... | ... | ... | ... | ... | — | ... | ... | ... | — | ... | ... | ... | ... | ... | ... | — | ... | — | ... | ... | ... | ... | ... |

### AFFECTIONS OF THE CHEST.

| THE CHEST. | Acon. | Ant. crud. | Arsenic. | Arnica. | Bella. | Bryonia. | Calc. | Carb. veg. | Capsicum. | Cham. | China. | Cina. | Cuprum. | Dulc. | Drosera. | Euphr. | Ferrum. | Hyos. | Ignat. | Ipecac. | Lachesis. | Nux vom. | Opium. | Puls. | Phosph. ac. | Rheum. | Rhus. | Samb. | Spigel. | Spongia. | Staph. | Stram. | Sulph. | Veratrum. |
|---|---|---|---|---|---|---|---|---|---|---|---|---|---|---|---|---|---|---|---|---|---|---|---|---|---|---|---|---|---|---|---|---|---|---|
| Determination of blood to the chest. | — | … | … | — | … | — | … | — | … | … | … | … | … | … | … | … | … | … | … | … | … | — | … | — | … | … | … | … | … | … | … | … | — | … |
| Burning in the chest | — | … | — | — | — | — | — | — | … | — | — | … | … | … | — | … | … | … | … | … | … | — | … | … | — | — | … | … | … | — | … | … | — | … |
| Pressure in do. | … | … | … | … | — | — | … | — | … | — | — | … | … | — | … | — | — | — | … | … | … | — | — | — | — | … | … | … | — | — | — | — | — | … |
| do. outward | … | … | … | … | … | — | … | … | … | … | … | — | … | … | … | … | … | … | … | … | … | … | … | … | … | … | … | … | … | … | … | … | … | … |
| do. inward | … | … | … | … | … | … | … | … | … | … | — | … | … | … | … | … | … | … | — | … | … | — | … | … | … | … | … | … | … | … | … | … | … | … |
| do. like a heavy weight | … | … | … | … | … | … | … | … | … | … | … | … |  | … | … | … | … | … | … | … | … | — | … | … | … | — | … | — | — | … | … | … | — | … |
| do. in the lower part of the chest | … | … | … | — | … | … | … | … | … | … | — | … | … | … | … | … | … | — | … | … | … | … | … | … | … | … | … | … | … | … | … | … | … | … |
| Pain | … | … | … | … | — | … | — | — | … | — | … | — | … | … | … | … | … | … | … | … | … | … | … | — | — | … | … | … | — | — | … | … | — | — |
| Feeling of tightness | … | … | — | … | — | … | … | — | … | … | … | … | … | — | … | … | … | … | … | … | … | … | … | — | — | … | … | … | — | … | … | … | — | … |
| Inflammation | — | … | … | — | … | — | … | … | … | … | … | … | … | … | … | … | … | — | … | … | … | — | … | — | … | … | — | … | … | — | … | — | … | … |
| Roughness in the chest | … | … | … | — | … | — | … | … | … | … | — | … | … | … | … | … | … | … | … | … | … | — | … | — | … | … | — | … | … | … | … | … | … | … |
| Cutting pain in the chest. | … | … | … | — | — | … | … | … | … | … | … | … | … | — | … | … | … | … | … | … | … | … | … | — | … | … | … | — | — | … | … | … | — | … |
| Stitches in the left side. | — | — | — | — | — | — | — | … | … | … | — | — | … | … | … | … | … | … | — | … | — | … | … | — | — | … | — | … | — | — | — | … | — | … |
| do. in the right side | — | … | … | — | … | — | — | — | … | … | — | … | … | — | … | … | … | — | — | … | — | … | — | … | — | … | — | … | … | … | … | … | — | … |
| do. in the middle | … | — | … | … | … | — | … | … | … | — | … | … | … | — | … | — | … | … | … | … | … | … | … | … | … | … | … | … | … | … | … | … | — | … |
| do. from within outward | … | … | … | … | … | — | … | … | … | … | — | … | … | … | … | … | … | … | … | … | … | … | … | … | … | … | … | … | — | — | … | … | … | … |
| Fullness | — | — | … | — | … | … | … | … | — | … | … | … | … | … | … | … | … | … | … | … | … | … | … | … | … | … | … | … | … | … | … | … | — | — |
| Hoarseness | — | … | — | … | — | — | — | — | — | — | — | … | — | … | — | … | — | … | … | … | — | — | … | — | — | … | — | … | … | — | — | … | — | — |
| Soreness in the chest | … | … | — | … | … | — | … | — | … | … | … | … | … | … | … | … | … | … | … | … | — | … | … | … | — | … | — | … | … | — | — | … | — | … |
| Palpitation of the heart | — | … | — | … | — | — | … | … | … | … | … | … | — | — | … | … | … | … | … | — | … | — | … | — | — | … | — | … | — | — | — | … | — | … |
| a) SYMPTOMS WORSE. | | | | | | | | | | | | | | | | | | | | | | | | | | | | | | | | | | |
| In the morning | … | — | … | — | … | — | … | … | … | … | — | — | … | … | … | … | … | … | — | … | … | — | … | — | … | … | … | … | … | … | — | — | … | … |
| In the afternoon | … | — | … | … | … | … | … | … | … | … | … | … | … | … | … | … | … | … | … | … | … | — | … | — | … | … | … | … | — | … | — | … | … | … |
| In the evening | … | … | — | — | … | — | … | … | … | … | — | … | … | … | … | … | — | … | — | … | … | … | … | — | … | … | — | … | — | … | … | … | … | … |
| At night | … | … | — | … | … | — | … | … | … | … | … | … | … | … | … | … | — | … | … | … | — | — | — | … | … | … | … | … | … | — | … | — | … | … |
| b) SYMPTOMS AGGRAVATED. | | | | | | | | | | | | | | | | | | | | | | | | | | | | | | | | | | |
| By breathing | — | … | — | … | … | — | — | … | — | … | … | … | … | — | … | … | … | … | … | … | … | … | … | — | … | … | … | … | … | … | … | … | … | … |
| By exhaling | … | … | … | … | … | — | … | … | … | … | — | … | … | … | … | … | … | … | … | … | … | … | … | … | — | … | … | … | — | … | — | … | … | … |
| By inhaling | — | … | … | … | … | — | — | … | … | — | — | … | … | … | … | … | … | … | … | … | … | … | — | — | — | … | — | … | — | — | … | … | … | … |
| By drawing a long breath | — | … | … | … | … | — | … | … | … | … | … | … | … | … | — | … | … | … | … | … | … | … | … | … | … | … | … | … | — | — | … | … | … | … |
| By touching | — | … | … | — | … | — | … | … | … | … | — | … | … | … | … | … | … | … | … | … | — | — | — | … | … | … | … | … | … | … | — | … | … | … |
| By moving | — | … | … | … | — | — | — | … | — | … | — | … | … | … | … | … | … | … | … | … | … | — | — | — | … | … | … | — | … | … | — | … | … | … |
| By stooping | — | … | … | — | … | — | — | … | … | … | — | … | … | … | … | … | … | … | … | … | … | — | … | — | — | … | … | … | … | … | — | … | … | … |
| By walking | — | … | … | … | … | — | … | … | … | … | … | … | … | … | … | … | … | … | … | … | … | — | … | … | … | … | — | … | — | … | — | … | … | … |
| After eating | … | … | … | … | … | — | … | … | … | … | — | … | … | … | … | … | … | … | — | … | … | — | … | … | … | … | … | … | … | … | … | … | … | … |
| In the open air | … | … | … | … | … | — | … | … | … | … | … | … | … | … | … | … | … | … | … | … | … | — | … | … | … | … | — | … | — | … | … | … | … | … |
| When coughing | — | — | — | — | … | — | — | — | … | … | … | … | … | … | … | … | — | … | … | — | … | — | … | — | … | … | … | … | … | … | … | … | — | … |
| When lying down | … | — | … | … | … | … | … | … | … | … | … | … | … | … | … | … | … | … | … | … | … | … | … | — | … | … | — | … | … | … | … | … | … | … |
| When lying on the left side | … | … | … | … | … | — | … | … | … | … | … | … | … | … | … | … | … | … | — | … | … | … | … | — | … | … | … | … | … | … | … | … | … | … |
| When lying on the right side | — | … | … | … | … | … | … | … | … | … | … | … | … | … | … | … | … | … | … | — | … | … | … | — | … | … | … | … | … | … | … | … | … | … |
| When lying on the painful side | … | … | … | … | — | … | … | … | — | … | … | … | … | … | … | … | … | … | … | … | … | — | … | … | … | … | … | … | … | — | … | … | … | … |
| When lying on the side not painful. | … | … | … | … | … | — | … | … | … | — | … | … | … | … | … | … | … | … | — | … | … | — | … | — | … | … | — | … | … | … | … | … | … | … |
| When sitting | … | … | … | … | … | … | … | — | … | … | — | … | … | … | — | … | — | … | … | … | … | … | … | — | … | … | — | … | — | — | — | … | … | … |
| c) SYMPTOMS RELIEVED. | | | | | | | | | | | | | | | | | | | | | | | | | | | | | | | | | | |
| By moving | … | … | … | — | … | … | … | … | … | … | … | … | … | … | — | … | … | … | … | … | … | … | … | … | … | … | … | … | … | … | … | … | … | … |
| By stooping | … | … | … | … | … | … | … | … | … | … | — | … | … | … | … | … | … | … | — | … | … | … | … | … | … | … | … | … | … | … | … | … | … | … |
| By external pressure | … | … | … | … | … | — | … | … | … | … | — | … | … | … | … | … | … | … | … | … | … | … | … | — | … | … | … | … | … | … | … | … | … | … |
| After eating | … | … | … | … | … | — | … | … | … | … | … | … | … | … | … | … | … | … | … | … | … | … | … | … | … | … | — | … | … | … | … | … | … | … |
| In the open air | … | … | … | … | … | — | … | … | … | … | … | … | … | … | … | … | … | … | … | … | … | … | … | — | … | … | … | … | … | … | … | … | … | … |
| When walking | … | … | … | … | … | … | … | … | … | … | — | … | … | … | — | … | … | … | … | … | … | … | … | — | … | … | — | … | … | … | … | … | … | … |
| When lying down | … | … | … | … | … | — | … | … | … | … | … | … | … | … | … | … | … | … | … | … | … | — | … | … | … | … | … | … | … | … | … | … | … | … |
| When lying on the back | … | … | … | … | … | — | … | … | … | … | … | … | … | … | … | … | … | … | — | … | … | … | … | — | … | … | … | … | … | … | … | … | … | … |
| When lying on the painful side | … | … | … | … | … | — | … | … | … | — | … | … | … | … | … | … | … | … | — | … | … | — | … | — | … | … | — | … | … | … | … | … | … | … |
| When lying on the side not painful. | … | … | … | … | … | … | … | … | … | … | … | … | … | … | … | … | … | … | — | … | … | — | … | … | … | … | … | … | … | … | … | … | … | … |

### COUGH AND ITS ATTENDANT SYMPTOMS.

| COUGHING. | Acon. | Arnica. | Arsenic. | Bell. | Bryonia. | Carb. Veg. | Calc. | Cham. | China. | Cina. | Cuprum. | Drosera. | Dulc. | Euphr. | Ferrum. | Hyos. | Ign. | Ipecac. | Lach. | Merc. viv. | Nux vom. | Opium. | Phosph. ac. | Puls. | Rhus tox. | Sambuc. | Spigel. | Spong. | Staph. | Sulph. | Veratrum. |
|---|---|---|---|---|---|---|---|---|---|---|---|---|---|---|---|---|---|---|---|---|---|---|---|---|---|---|---|---|---|---|---|
| With expectoration | ... | — | — | ... | — | — | — | — | — | — | ... | — | ... | — | ... | ... | ... | — | ... | ... | — | ... | — | — | ... | ... | — | — | — | — | ... |
| With easy expectoration | ... | ... | ... | ... | ... | ... | ... | ... | ... | ... | ... | ... | — | — | ... | ... | ... | ... | ... | ... | ... | ... | ... | — | ... | ... | ... | ... | ... | ... | ... |
| With difficult expectoration | ... | ... | ... | ... | — | ... | ... | — | ... | — | ... | — | ... | — | ... | — | ... | ... | — | ... | — | ... | ... | — | ... | ... | ... | ... | — | ... | ... |
| Dry cough | — | — | — | — | — | — | — | — | ... | ... | — | ... | ... | ... | ... | — | — | — | ... | — | — | — | — | ... | — | ... | ... | — | ... | — | — |
| Barking cough | ... | ... | ... | ... | ... | ... | ... | ... | ... | ... | ... | — | — | ... | ... | ... | ... | ... | ... | ... | ... | ... | ... | ... | ... | ... | ... | — | ... | ... | ... |
| Suffocating cough | — | ... | ... | ... | — | ... | ... | — | ... | ... | — | ... | ... | ... | ... | ... | ... | — | ... | ... | ... | — | ... | ... | ... | ... | — | — | ... | ... | ... |
| Hollow cough | — | ... | ... | ... | ... | ... | ... | ... | ... | — | ... | ... | ... | ... | ... | ... | ... | ... | ... | ... | ... | — | ... | ... | ... | — | — | — | ... | ... | — |
| Hoarse cough | — | ... | ... | ... | ... | ... | ... | ... | — | — | ... | — | ... | ... | ... | ... | ... | ... | ... | ... | ... | ... | ... | ... | ... | — | ... | — | ... | ... | — |
| Hooping cough | — | ... | ... | ... | — | — | ... | — | — | — | — | — | — | — | ... | — | ... | ... | ... | ... | — | ... | ... | — | ... | ... | ... | — | ... | ... | — |
| Croupy cough | — | ... | ... | — | ... | ... | ... | — | — | — | ... | — | ... | ... | ... | — | ... | ... | ... | ... | — | ... | ... | ... | ... | — | ... | — | ... | ... | — |
| Spasmodic cough | — | ... | ... | — | — | — | ... | ... | — | — | ... | ... | ... | ... | ... | — | ... | — | ... | ... | — | ... | ... | — | ... | ... | ... | ... | — | ... | ... |
| Short cough | — | ... | ... | ... | ... | ... | ... | ... | ... | ... | ... | ... | ... | ... | ... | ... | — | ... | ... | ... | — | ... | ... | — | — | ... | ... | — | ... | ... | ... |
| Continual cough | ... | ... | ... | ... | ... | ... | ... | ... | ... | ... | — | — | ... | ... | ... | — | ... | — | ... | ... | ... | ... | ... | ... | ... | ... | ... | ... | ... | ... | ... |
| a) EXPECTORATION. | | | | | | | | | | | | | | | | | | | | | | | | | | | | | | | |
| Bitter | ... | ... | — | ... | ... | ... | ... | — | ... | ... | ... | — | ... | ... | ... | ... | ... | ... | ... | ... | ... | ... | ... | — | ... | ... | ... | ... | ... | ... | ... |
| Intermixed with blood | ... | — | — | — | — | ... | ... | ... | ... | — | ... | ... | — | ... | — | — | ... | ... | — | — | ... | ... | ... | — | ... | ... | ... | ... | ... | — | ... |
| Coagulated blood | ... | — | ... | ... | — | ... | ... | ... | ... | ... | ... | ... | ... | ... | ... | ... | ... | ... | ... | ... | — | ... | ... | — | ... | ... | ... | ... | ... | ... | ... |
| Yellow | ... | ... | — | ... | — | ... | — | ... | ... | ... | ... | — | ... | ... | ... | ... | — | ... | ... | ... | ... | ... | — | — | ... | ... | ... | — | — | — | ... |
| Green | ... | ... | ... | ... | ... | ... | — | ... | ... | ... | ... | ... | ... | ... | — | — | ... | ... | ... | ... | ... | ... | ... | — | ... | ... | ... | ... | ... | — | ... |
| Abundant | ... | ... | ... | ... | — | ... | — | ... | ... | — | ... | — | ... | ... | ... | ... | ... | ... | ... | ... | ... | ... | ... | — | ... | ... | ... | ... | ... | — | ... |
| Tasting saltish | ... | ... | — | ... | ... | ... | ... | ... | ... | ... | ... | ... | ... | ... | ... | ... | ... | ... | ... | ... | ... | ... | ... | — | ... | ... | ... | ... | ... | ... | — |
| Tasting sweet | ... | ... | ... | ... | ... | ... | — | ... | ... | ... | ... | ... | ... | ... | — | ... | ... | ... | ... | ... | — | ... | ... | ... | ... | ... | ... | ... | ... | ... | ... |
| Slimy | ... | ... | — | — | — | ... | — | — | — | — | ... | ... | ... | ... | ... | — | ... | ... | ... | ... | ... | — | ... | — | — | ... | ... | — | — | — | ... |
| b) SYMPTOMS WORSE. | | | | | | | | | | | | | | | | | | | | | | | | | | | | | | | |
| In the morning | ... | — | — | ... | — | ... | ... | ... | — | ... | ... | ... | ... | — | — | ... | ... | ... | ... | ... | — | ... | ... | — | — | ... | ... | ... | ... | ... | — |
| In the forenoon | ... | ... | ... | — | — | ... | ... | ... | ... | ... | ... | ... | ... | ... | ... | ... | ... | ... | ... | ... | ... | ... | ... | ... | ... | ... | ... | ... | — | ... | ... |
| In the afternoon | ... | ... | ... | ... | — | ... | ... | ... | ... | ... | ... | ... | ... | ... | ... | ... | ... | ... | ... | ... | ... | ... | ... | — | ... | ... | ... | ... | — | ... | ... |
| In the evening | ... | ... | — | ... | — | — | — | ... | ... | — | ... | — | ... | ... | ... | ... | — | ... | ... | ... | — | ... | ... | — | — | ... | ... | ... | ... | — | — |
| At night | ... | — | — | — | — | — | — | ... | ... | ... | ... | ... | — | ... | — | — | ... | ... | — | — | — | ... | ... | — | — | ... | ... | ... | ... | — | — |
| Before midnight | ... | ... | ... | ... | ... | — | — | ... | ... | ... | ... | ... | ... | ... | — | ... | ... | ... | ... | ... | — | ... | ... | ... | — | ... | ... | ... | ... | ... | ... |
| After midnight | — | ... | ... | ... | — | ... | ... | ... | — | ... | ... | — | ... | ... | ... | ... | ... | ... | ... | ... | — | ... | ... | ... | — | ... | ... | ... | ... | ... | ... |
| c) COUGH CAUSED. | | | | | | | | | | | | | | | | | | | | | | | | | | | | | | | |
| By overheating | — | ... | ... | ... | — | ... | ... | ... | ... | ... | ... | ... | ... | ... | ... | ... | ... | — | ... | ... | — | ... | ... | ... | ... | ... | ... | ... | ... | ... | ... |
| By taking cold | ... | ... | ... | ... | — | — | ... | ... | ... | ... | — | — | — | ... | ... | ... | ... | — | ... | ... | — | ... | ... | ... | — | ... | ... | ... | ... | ... | ... |
| By drawing a long breath | ... | ... | ... | ... | — | ... | ... | ... | ... | — | ... | ... | — | ... | ... | ... | ... | — | ... | ... | ... | ... | ... | ... | ... | ... | ... | ... | ... | ... | ... |
| By moving | ... | — | ... | ... | — | ... | ... | ... | ... | ... | ... | ... | ... | ... | ... | ... | ... | ... | ... | ... | — | ... | ... | ... | ... | ... | ... | ... | ... | ... | ... |
| By irritation in the chest | ... | — | ... | ... | ... | ... | ... | — | ... | ... | ... | ... | ... | ... | ... | ... | ... | ... | ... | ... | ... | ... | ... | ... | ... | ... | ... | — | ... | ... | — |
| By slime in the chest | ... | ... | ... | ... | ... | ... | ... | — | ... | — | ... | ... | ... | ... | ... | ... | ... | ... | ... | ... | ... | ... | ... | — | ... | ... | ... | ... | ... | ... | ... |
| By stitches in the chest | — | ... | ... | ... | — | ... | — | ... | ... | ... | ... | ... | ... | ... | ... | ... | ... | ... | — | ... | — | ... | ... | ... | ... | ... | ... | ... | ... | — | ... |
| By being in the open air | — | ... | ... | ... | — | ... | ... | ... | ... | ... | ... | ... | ... | ... | ... | ... | ... | — | ... | ... | — | ... | ... | ... | ... | ... | — | — | ... | ... | ... |
| By mental emotions | — | — | ... | ... | — | ... | ... | ... | ... | ... | ... | ... | ... | ... | ... | ... | ... | ... | ... | ... | — | — | ... | ... | — | ... | ... | ... | — | ... | ... |
| By tickling in the throat | ... | ... | ... | — | — | ... | ... | ... | — | ... | ... | ... | ... | ... | ... | ... | ... | ... | — | — | ... | ... | — | ... | ... | ... | ... | ... | ... | — | ... |
| By roughness in the throat | ... | ... | ... | ... | — | ... | ... | ... | ... | ... | ... | ... | ... | ... | ... | ... | ... | ... | ... | ... | ... | ... | ... | ... | ... | ... | ... | ... | ... | ... | ... |
| By dryness in the throat | ... | ... | ... | ... | — | ... | ... | ... | ... | ... | ... | — | ... | ... | ... | ... | ... | ... | ... | ... | ... | ... | ... | — | — | ... | ... | ... | ... | ... | ... |
| By irritation in the windpipe | — | — | ... | ... | — | ... | ... | ... | ... | ... | ... | — | ... | ... | ... | ... | — | — | ... | ... | — | ... | ... | ... | ... | ... | ... | ... | — | ... | ... |
| By drinking | — | ... | — | ... | — | ... | ... | ... | ... | ... | ... | ... | ... | ... | — | ... | ... | ... | ... | ... | — | ... | ... | ... | ... | ... | ... | ... | ... | ... | ... |
| By nausea | ... | ... | ... | ... | ... | ... | ... | ... | ... | ... | ... | ... | ... | ... | ... | ... | ... | ... | ... | ... | — | ... | ... | ... | ... | ... | ... | ... | ... | ... | ... |
| Before rising | ... | ... | ... | ... | ... | ... | ... | ... | ... | ... | ... | ... | ... | ... | ... | ... | ... | ... | ... | ... | — | ... | ... | ... | ... | ... | ... | ... | ... | ... | ... |
| After eating | ... | ... | ... | ... | — | ... | ... | ... | — | ... | ... | ... | ... | ... | — | ... | ... | ... | — | ... | — | ... | ... | ... | ... | ... | ... | ... | — | ... | ... |
| When lying down | ... | ... | ... | ... | ... | ... | ... | ... | ... | ... | ... | ... | ... | ... | — | — | — | ... | ... | ... | — | ... | ... | — | ... | ... | ... | ... | ... | — | ... |
| When lying on the back | ... | ... | ... | ... | ... | ... | ... | ... | ... | ... | ... | ... | ... | ... | ... | ... | ... | ... | ... | ... | — | ... | ... | ... | ... | ... | ... | ... | ... | ... | ... |
| When lying on the left side | — | ... | ... | ... | — | ... | ... | ... | ... | ... | ... | ... | ... | ... | ... | ... | ... | — | ... | ... | ... | ... | ... | — | ... | ... | ... | ... | ... | ... | ... |
| When lying on the right side | — | ... | ... | ... | ... | ... | ... | ... | ... | ... | ... | ... | ... | ... | ... | ... | ... | — | ... | ... | ... | ... | ... | ... | ... | ... | ... | ... | ... | ... | ... |
| While being quiet | ... | ... | ... | ... | ... | ... | ... | ... | ... | ... | ... | — | ... | ... | — | — | ... | ... | ... | ... | — | ... | ... | — | ... | ... | ... | ... | — | ... | ... |
| After lying down | ... | ... | ... | ... | ... | ... | ... | ... | ... | ... | ... | ... | — | ... | ... | ... | ... | ... | ... | ... | — | ... | ... | — | — | ... | ... | ... | — | ... | ... |

